# Cyclic Behavior of Reinforced High Strain-Hardening UHPC under Axial Tension

**DOI:** 10.3390/ma14133602

**Published:** 2021-06-28

**Authors:** Jin-Ben Gu, Jun-Yan Wang, Yi-Qing Guo

**Affiliations:** 1 Key Laboratory of Advanced Civil Engineering Materials, Ministry of Education, Tongji University, Shanghai 201804, China; jb.gu@tongji.edu.cn (J.-B.G.); guoyiqing1989@163.com (Y.-Q.G.); 2 Department of Structural Engineering, Tongji University, Shanghai 201804, China

**Keywords:** cyclic tension, high strain-hardening UHPC, steel reinforcement, tension-stiffening effect, stiffness degradation mechanism

## Abstract

The cyclic tensile behavior of steel-reinforced high strain-hardening ultrahigh-performance concrete (HSHUHPC) was investigated in this paper. In the experimental program, 12 HSHUHPC specimens concentrically placed in a single steel reinforcement under cyclic uniaxial tension were tested, accompanied by acoustic emission (AE) source locating technology, and 4 identical specimens under monotonic uniaxial tension were tested as references. The experimental variables mainly include the loading pattern, the diameter of the embedded steel rebar, and the level of target strain at each cycle. The tensile responses of the steel-reinforced HSHUHPC specimens were evaluated using multiple performance measures, including the failure pattern, load–strain response, residual strain, stiffness degradation, and the tension-stiffening behavior. The test results showed that the enhanced bond strength due to the inclusion of steel fibers transformed the failure pattern of the steel-reinforced HSHUHPC into a single, localized macro-crack in conjunction with a sprinkling of narrow and closely spaced micro-cracks, which intensified the strain concentration in the embedded steel rebar. Besides, it was observed that the larger the diameter of the embedded steel rebar, the smaller the maximum accumulative tensile strain under cyclic tension, which indicated that the larger the diameter of the embedded steel rebar, the greater the contribution to the tensile stiffness of steel-reinforced HSHUHPC specimens in the elastic–plastic stage. In addition, it was found that a larger embedded steel rebar appeared to reduce the tension-stiffening effect (peak tensile strength) of the HSHUHPC. Moreover, the residual strain and the stiffness of the steel-reinforced HSHUHPC were reduced by increasing the number of cycles and finally tended toward stability. Nevertheless, different target strain rates in each cycle resulted in different eventual cumulative tensile strain rates; hence the rules about failure pattern, residual strain, and loading stiffness were divergent. Finally, the relationship between the accumulative tensile strain and the loading stiffness degradation ratio under cyclic tension was proposed and the tension-stiffening effect was analyzed.

## 1. Introduction

Ultrahigh-performance concrete (UHPC), as one of the current advanced high-performance cementitious composites, exhibits eminent mechanical properties: comparatively high compressive strength (≥120 MPa) and tensile strength (≥10 MPa), strain-hardening characteristics under tensile load for a given volume of discontinuous internal fibers, and excellent durability due to an optimized dense matrix as well [1,2,3]. Besides, in accordance with the UHPC design guidelines issued by the Federal Institute of Technology in Lausanne, Switzerland [4], UHPC can be categorized into three types: UO (strain softening), UA (ultimate tensile hardening strain is higher than 1500 με), and UB (ultimate tensile hardening strain is higher than 2000 με). Distinctly, the ultimate tensile hardening strain of the UB type is usually higher than the yield strain of the normal steel rebar, typically about 2000 με. The high strain-hardening UHPC (HSHUHPC) defined herein refers to the UB type of UHPC. In previous studies, it has been shown that the HSHUHPC has excellent crack width control ability, superior strength, ductility, and excellent durability [5].

The intensified properties of UHPC have urged a wide range of researchers and engineers to apply it in various types of innovative civil engineering, where bending prevails [6]. Nevertheless, since the tensile performance of UHPC mainly depends on fiber orientation and distribution to a large extent, it is proposed that steel rebars are configured in UHPC to further provide a significant improvement in structural behavior for steel-reinforced UHPC components in practical applications [7]. Hence the variation in tensile behavior of UHPC is reduced due to the randomness of fiber orientation and distribution when steel rebars are configured. In addition, the inclusion of fibers in steel-reinforced UHPC members effectively increases the bond performance between the reinforcement and surrounding UHPC due to the enhanced tensile fracture energy of the UHPC [8]. Moreover, steel-reinforced UHPC might have a positive cost effect because of the massive reduction in the quantity of expensive steel fibers [9].

**Figure 1:**
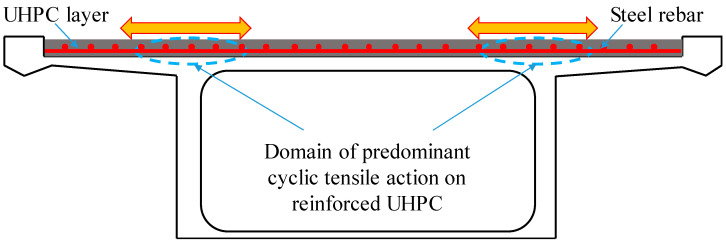
Bridge decks strengthened with a thin reinforced UHPC overlay.

Recently, the rehabilitation of a concrete bridge deck or orthotropic steel deck was widely conducted by employing a thin reinforced UHPC overlay, instead of a relatively thicker reinforced concrete layer, because the demand to upgrade the load-bearing capacity of bridge structures is rising [10,11]. In these scenarios, the thin reinforced UHPC layer is often cast in a state of cyclic tensile stress on the negative moment region, which is subjected to repeating wheel loads, and the thin reinforced UHPC layer behaves as a direct tension member, as shown in Figure 1. This phenomenon also exists in the negative bending moment of the steel–UHPC composite bridge deck system. However, the tensile behavior of reinforced UHPC is yet to be fully understood, especially for the cyclic tensile behavior of reinforced UHPC. Several researchers have investigated the static behavior of reinforced UHPC under tension [12,13]. Minghong Qiu et al. [14] investigated the effects of reinforcement ratio, fiber orientation, and fiber chemical treatment on the tensile behavior of reinforced UHPC specimens. The test results demonstrated that in the case of fiber chemical treatment with ZnPh, it has no noticeable effect on the direct tensile performance of rebar-reinforced UHPC specimens. T. Redaelli et al. [15] conducted direct tension tests on real-scale (160 mm × 160 mm cross section with 1 m gauge length) UHPC dog bone-shaped specimens reinforced with ordinary steel rebars (16 mm diameter). The test results manifested that the cracks opening at the serviceability-limit state were thin and closely spaced (spacing of 20–100 mm). Moreover, the tension-stiffening effect in reinforced UHPC was more pronounced than that in reinforced concrete (RC) members, resulting in a higher stiffness of the composite. Chung-Chan Hung et al. [16,17] performed direct tensile tests of reinforced UHPC specimens, with the experimental variables including embedded rebar sizes, loading patterns, and steel fibers. The test results showed that the inclusion of a 2% volume fraction of fibers substantially improved the tensile stiffness and strength of the reinforced UHPC specimens with reinforcing ratios between 0.88% and 2.25% as well. Roy et al. [18] studied the effect of fiber content, fiber orientation, and steel rebar type on the tensile behavior of reinforced UHPC. The test results demonstrated higher fiber content, better fiber orientation, and higher strength grade of steel rebars. This further resulted in the higher tensile strength of rebar-reinforced UHPC. Makita et al. [19,20] conducted tensile fatigue tests on R-UHPFRC. The experimental results showed a fatigue endurance limit at 10 million cycles at a solicitation level of S = 0.54, S being the ratio between the maximum fatigue force and the ultimate strength. In summary, most studies have focused on the monotonic tensile properties of reinforced UHPC; thus, it is necessary to study the direct tensile behavior of reinforced UHPC members under cyclic loading, which is conducive to understanding and assessing the structural performance of bridge decks strengthened with a thin reinforced UHPC layer or the steel–UHPC composite deck under repeated wheel loads.

The objective of the present study was to investigate the behavior of steel-reinforced HSHUHPC members under cyclic axial tension at the serviceability-limit state. Twelve steel-reinforced HSHUHPC dog-bone-shaped specimens under cyclic uniaxial tension and four identical specimens under monotonic uniaxial tension were fabricated and tested, supplemented with crack width detection and AE source locating technology as well. AE technology provides support in detecting the internal damages of reinforced UHPC from the microcosmic point of view. AE source locating is a method to obtain AE source distribution in three-dimensional space. The experimental variables included the loading pattern, diameter of embedded steel rebar, and the level of target strain at each cycle. All of the specimens were loaded for 10 cycles. The evolution of mechanical properties, damage mode, crack spacing, distribution of damage points, residual strain, tension-stiffening response, and stiffness degradation mechanism of steel-reinforced HSHUHPC members under cyclic tension were investigated and analyzed.

## 2. Experimental Program

### 2.1. Description of Test Specimens

**Figure 2:**
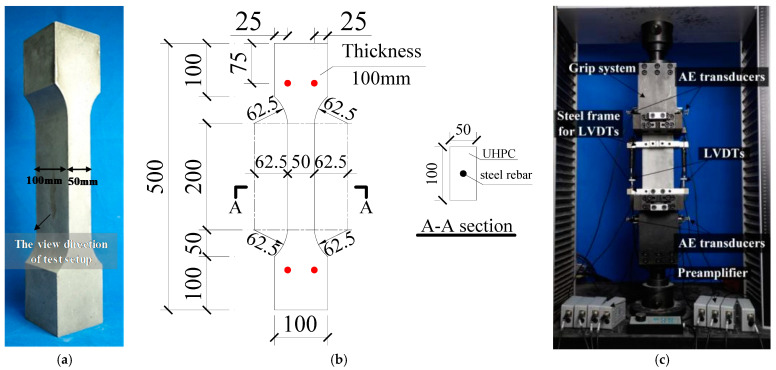
Direct tensile test system with AE analysis technology: (**a**) specimen; (**b**) dimensions and reinforcement details; (**c**) test setup.

A total of 12 steel-reinforced HSHUHPC dog-bone-shaped specimens under cyclic uniaxial tension and four specimens under monotonic uniaxial tension as reference specimens, with identical dimensions, were fabricated and tested. The detailed dimensions of all specimens are shown in Figure 2. The experimental variables mainly include the loading pattern (monotonic loading or cyclic loading), the diameter of the embedded rebar (10 mm, 12 mm, 14 mm, and 16 mm), and the level of target strain at each cycle (1000 με, 2000 με, and 2500 με). The steel rebars with a diameter of 10 mm, 12 mm, 14 mm, and 16 mm were equivalent to the reinforcement ratios of 1.57%, 2.26%, 3.08%, and 4.02%, respectively. It could be seen that the reinforcement ratio of steel-reinforced HSHUHPC is relatively high compared with that of RC members. Besides, three levels of target strain correspond to the different working states of steel-reinforced HSHUHPC structures. In the case that the target strain for each cycle is 1000 με or 2000 με, the steel rebar is in the elastic state, while HSHUHPC is in the strain hardening state. Additionally, in the case that the target strain for each cycle is 2500 με, the steel rebar is in the yielding state, while HSHUHPC is in the strain hardening state as well. The notations and the design details for all the tested specimens are summarized in Table 1. For cyclic tensile specimens, each specimen is denoted as “diameter of steel rebar (D-)” and “the level of target strain.” For example, D10-1000 refers to the HSHUHPC specimen reinforced with a steel rebar with a diameter of 10 mm, and tested under cyclic tensile loading corresponding to a target strain of 1000 με at each cycle. Besides, during the casting process, six cubic specimens (100 mm × 100 mm × 100 mm) and three dog-bone-shaped HSHUHPC specimens with no reinforcement were retained to obtain the axial compressive and axial tensile properties of HSHUHPC. Similarly, three rebars of each diameter are retained to obtain the stress–strain curve of the pure steel rebars.

### 2.2. Materials

#### 2.2.1. UHPC

**Figure 3:**
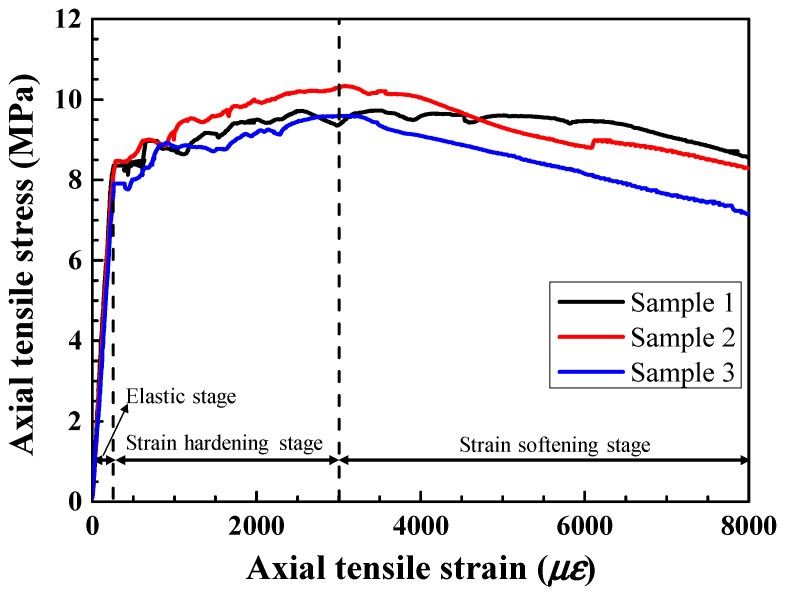
Axial tensile stress–strain curves of HSHUHPC.

The HSHUHPC materials used in this research consist of UHPC premixed powder, steel fibers, and water. Table 2 provides the mix proportions of the HSHUHPC matrix. A type of smooth straight steel fiber with brass coating was used, whose properties are given in Table 3. The volume fraction of smooth straight steel fibers used in HSHUHPC is 1.8%. Additionally, all the cube specimens and dog-bone-shaped specimens were cured under the non-steam cured condition. The axial compressive strength of HSHUHPC was obtained according to the Chinese standard GB/T 31387 2015 [21] by using a universal testing machine with an ultimate load capacity of 3000 kN. The 28-day average compressive strength (three specimens) of HSHUHPC is 154.1 MPa with a standard deviation of 3.86 MPa. Besides, the axial tensile stress–strain curves of three dog-bone-shaped HSHUHPC samples derived from the direct tensile test are illustrated in Figure 3. As shown in Figure 3, HSHUHPC obtained the ultimate tensile strength of around 9.77 MPa and the ultimate tensile strain of around 3000 με.

#### 2.2.2. Steel Rebar

**Figure 4:**
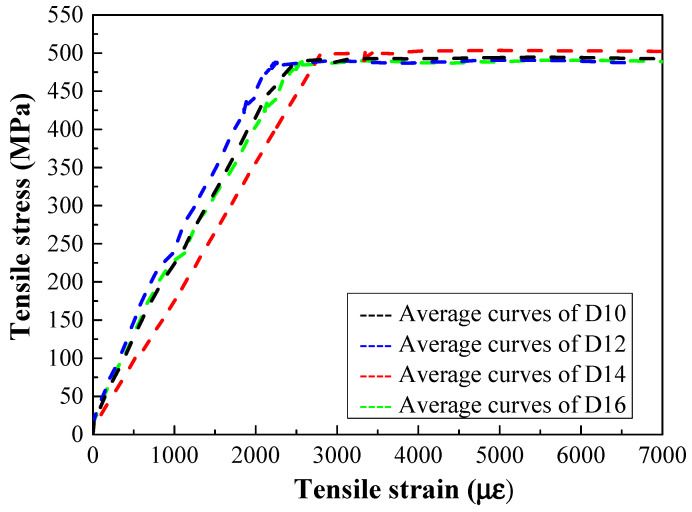
Axial tensile stress–strain curves of HSHUHPC.

For steel-reinforced HSHUHPC specimens, HRB400 deformed steel rebars with different diameters and a length of 500 mm were used for longitudinal reinforcement. Three bare steel rebars were tested to obtain the tensile stress–strain characteristics. The average tensile stress–strain relationship of the steel rebars, which were tested in a universal testing machine, was obtained as shown in Figure 4. All the steel rebars used in this research have a yield strength of around 460 MPa with a standard deviation of 6 MPa and a yield strain of around 2500 με.

### 2.3. Test Setup, Instrumentation and Test Procedure

A direct tensile test was carried out through a universal testing machine (WDW-300 servo-controlled testing system) running in a displacement control manner. The configuration, dimensions, and test setup of the steel-reinforced HSHUHPC dog-bone specimens are presented in Figure 2. Moreover, a direct tensile test, supplemented with crack width detection and AE source locating technology as shown in Figure 2c as well, was conducted to study the tensile damage mechanism of steel-reinforced HSHUHPC under cyclic tension, whose test setup is illustrated in Figure 2 as well, in which: (1) a dog-bone-shaped specimen as shown in Figure 2a was fabricated in accordance with the dimension details of Figure 2b; (2) a set of customizing fixtures was used to avoid secondary flexural stress and to ensure a centric loading condition as shown in Figure 2; and (3) two linear variable differential transformers (LVDTs), whose gauge length was 150 mm, were mounted on the specimen to measure the tensile elongation. In addition, eight AE transducers were placed in a rectangular array just above the surface of the double sides of the tensile specimens to pick up AE signals originating from the specimens, in parallel with the direct tensile test as shown in Figure 2c.

### 2.4. Loading Scheme

Four loading scenarios were performed in this study. Before each test, a preloading of 0.5 kN was applied and was then unloaded to zero in order to stabilize the testing system and obtain the complete stress–strain curve originating from zero point. For the monotonic loading scheme, the specimens were subjected to a displacement control load with a speed of 0.3 mm/min until the specified strain. For cyclic loading schemes, three different loading histories were used to investigate the damage evolutions of steel-reinforced HSHUHPC under cyclic tension with three levels of target strains (1000 με, 2000 με, and 2500 με) corresponding to the different service states of reinforced UHPC structures. In the case of a given target strain, the loading part of each cycle was performed when the preloading force reached 0.5 kN, while after attaining the target strain, the unloading part was started and finished until 0.5 kN. What calls for special attention is that for the *i* + 1 cycle; the total strain amplitude of the *i* + 1 cycle is the superposition of the cumulative residual strain generated on the *i* cycle and the target strain value. Moreover, the loading rate remained at a speed of 0.3 mm/min throughout the whole loading process, whether for the loading part or the unloading part. All the specimens were loaded for 10 cycles.

## 3. Test Results

### 3.1. Failure Pattern

The test results implied that despite significantly different reinforcement ratios and loading patterns, the level of target strain in the case of cyclic tension led to a distinct difference in the failure pattern of steel-reinforced HSHUHPC members caused by various eventual accumulative ultimate strains. When the level of target strain was 1000 με, after 10 cycles, all the specimens exhibited multiple micro-cracks invisible to the naked eye. It is worth noting that the boundary of the crack width is 0.05 mm between the invisible micro-crack and the visible macro-crack. This also demonstrates that the HSHUHPC possesses good control ability in terms of crack width.

**Figure 5:**
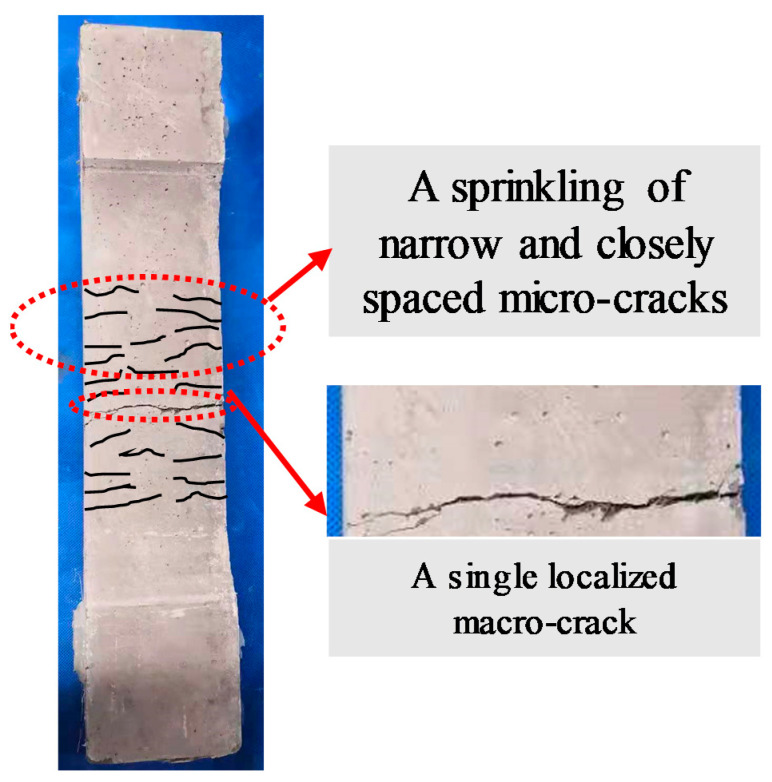
The typical failure mode of specimen D12-2000.

When the levels of target strain for the tested specimens are 2000 με and 2500 με, after 10 cycles, the maximum tensile strain generally has exceeded the yield strain of the reinforcement and ultimate tensile strain of HSHUHPC. The damage pattern is transformed into a single macro-crack along with a series of narrow and closely spaced micro-cracks. The main reason is that the tensile strain-hardening characteristics of the HSHUHPC provide the steel-reinforced HSHUHPC members with an effective crack width control ability for the initially opened multiple micro-cracks prior to the yielding of the embedded steel rebar. Once the embedded steel rebar begins to yield, the gradually increased demand for deformation on the specimen progressively results in the occurrence and propagation of a localized macro-crack at the weakest cross section, possibly where the least effective number of fibers are distributed in the direction parallel to the tension loading; however, the local strain in the other segment is still below the ultimate strain. Eventually, there is a pronounced post-yield localized macro-crack. It is worth noting that the occurrence of the visible macro-crack was between the yielding strain of the steel rebar and the ultimate tensile strain of HSHUHPC, that is, around 2700–2840 με. This may be due to the fact that as the steel rebar yields, the steel fibers at the crack are motivated to debond and pull out gradually and the synergistic effect between the steel rebar and HSHUHPC weakens. Besides, the steel fibers at the crack cannot balance the large deformation demands of the steel rebar after yielding, despite the presence of the bridging effect of the steel fibers. Figure 5 shows the typical crack patterns of specimen D12-2000.

### 3.2. Load–Strain Relationship

**Figure 6:**
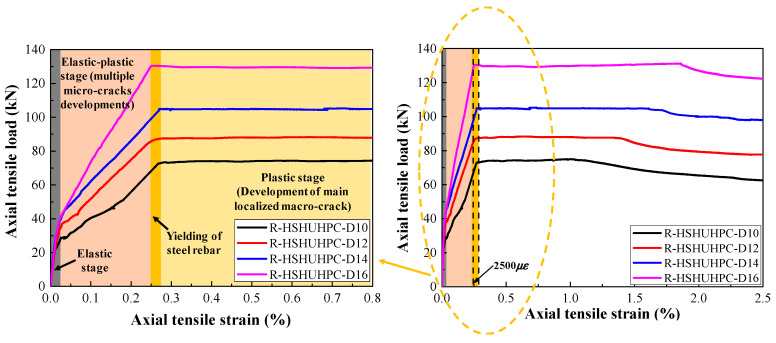
The tensile load–strain response of steel-reinforced HSHUHPC under monotonic uniaxial tension.

**Figure 7:**
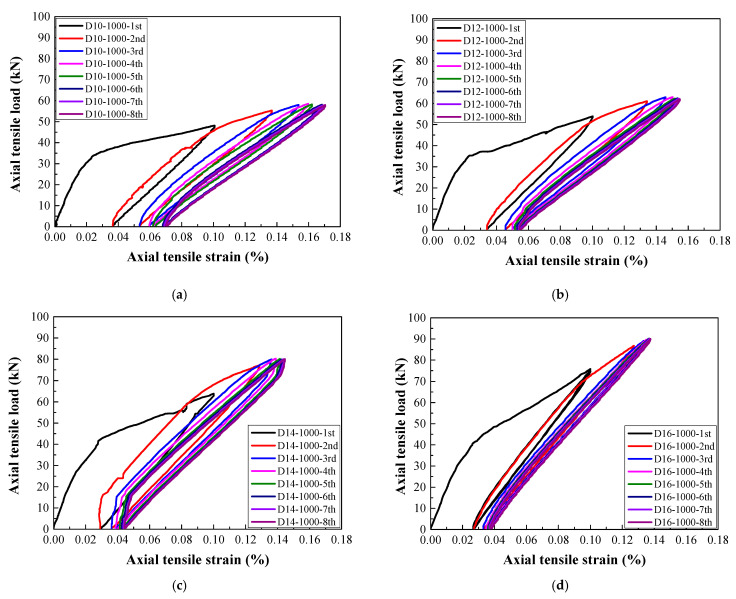
The load–strain response of steel-reinforced HSHUHPC specimens with a specified target strain of 1000 for different diameters: (**a**) 10 mm; (**b**) 12 mm; (**c**) 14 mm; (**d**) 16 mm.

**Figure 8:**
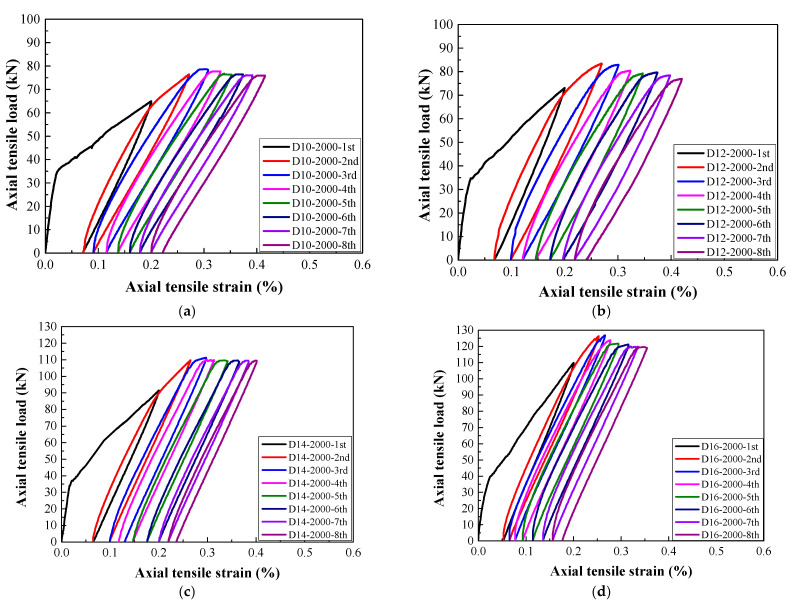
The load–strain response of R-HSHUHPC specimens with a specified target strain of 2000 for different diameters: (**a**) 10 mm; (**b**) 12 mm; (**c**) 14 mm; (**d**) 16 mm.

**Figure 9:**
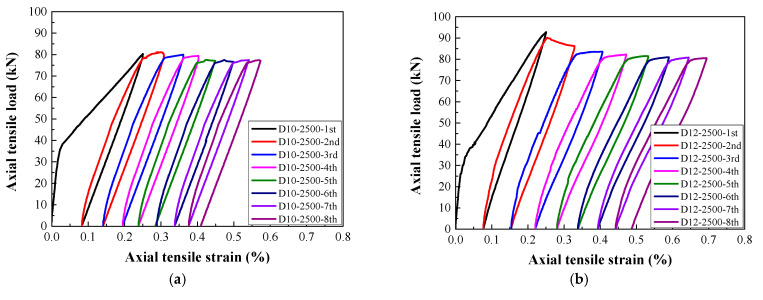

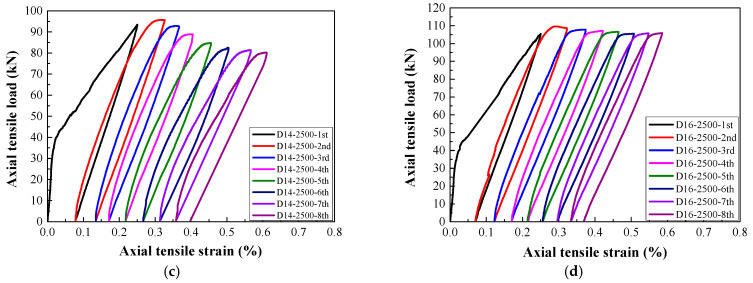
The load–strain response of steel-reinforced HSHUHPC specimens with a specified target strain of 2500 for different diameters: (**a**) 10 mm; (**b**) 12mm; (**c**) 14 mm; (**d**) 16 mm.

Figure 6, Figure 7, Figure 8 and Figure 9 present the response of the tensile load versus tensile strain for the steel-reinforced HSHUHPC members under monotonic and cyclic tension, respectively. It can be observed from Figure 6 that in the case of monotonic tension, the tensile load–strain curve for the steel-reinforced HSHUHPC specimens consists of three stages, i.e., elastic stage, elastic–plastic stage (development of multiple micro-cracks), and plastic stage (development of the main localized macro-crack). In the elastic stage, all the specimens behaved as elastically linear between the tensile load and tensile strain before the applied load reached the cracking load. With a progressively increasing tensile load, the slope of the tensile load–strain curve smoothly decreased, revealing the reduction of the specimen’s tensile stiffness. The specimen accessed the stage of multiple invisible micro-crack development along with the augment in the number and width of closely spaced micro-cracks. Here, it is noticed that the curve segment in the stage of multiple invisible micro-crack development was almost parallel to that of the pure steel rebar, different from that of the normal concrete or fiber-reinforced concrete. This might be due to the fact that the strain-hardening characteristic of HSHUHPC resulted in a great improvement in stiffness. As the applied load was up to the plastic stage, the steel rebar began to yield, and the weakest one of the multiple invisible micro-cracks was propagated into a single localized crack, and the crack width of the main localized crack developed rapidly. Taken together, under the monotonic axial tension, it appeared that steel-reinforced HSHUHPC members failed at a lower overall tensile strain compared with the RC structures, due to the higher bond behavior between HSHUHPC and the steel rebar, and the single localized macro-cracks exacerbated the strain concentration in the embedded steel rebar. 

As shown in Figure 7, Figure 8 and Figure 9, the typical tensile load–strain curves of all steel-reinforced HSHUHPC under cyclic tension could be divided into two types according to the different types of damage pattern. When the level of the target strain is 1000 με, the tensile load–strain curves could be divided into stage I (the elastic stage) and stage II (the elastic–plastic stage), and both stages displayed a smooth transition. Furthermore, in the elastic–plastic stage, the tensile stiffness of the specimens progressively reduced, and the slope of the curve was slightly less than that in the elastic stage. Additionally, for the 1st cycle, the load–strain curves generally consisted of the elastic loading stage, strain hardening stage, and unloading stage. Additionally, for the 2nd to 4th cycles, the load–strain curves consist of the nonlinear loading stage, strain hardening stage, and unloading stage. All the load–strain curves from the 5th to 8th cycles were approximately coincidental and exhibited stable closed rings, which only consisted of a nonlinear loading part and an unloading part. Besides, the slopes for reloading and unloading both decreased to a stable value, revealing that the cumulative damage of the steel-reinforced HSHUHPC did not continue to develop with the addition in the number of cycles. When the levels of the target strain are 2000 με and 2500 με, the tensile load–strain curves could be divided into stage I (the elastic stage), stage II (the elastic–plastic stage), and stage III (the plastic stage). It was observed that the strain at the beginning of stage III was approximately the same as the yield strain of the HRB400 steel rebar (around 2500 με). Thus, it was supposed that there was hardly a slip generated between HSH-UHPC and the steel rebar before the yielding of the steel rebar. Besides, for the 1st cycle, the load–strain curves generally consisted of the elastic loading part, strain hardening part, and unloading part, which was identical to that of the level of the target strain of 1000 με. After the 2nd or 3rd cycle, the load–strain curves all consisted of the nonlinear loading part, strain hardening part, and unloading part due to the yielding of the embedded steel rebar. What calls for special attention is that the cumulative residual strain decreases gradually due to the increased axial tensile stiffness as the diameter of the embedded steel rebar increases.

### 3.3. Envelope Curves

**Figure 10:**
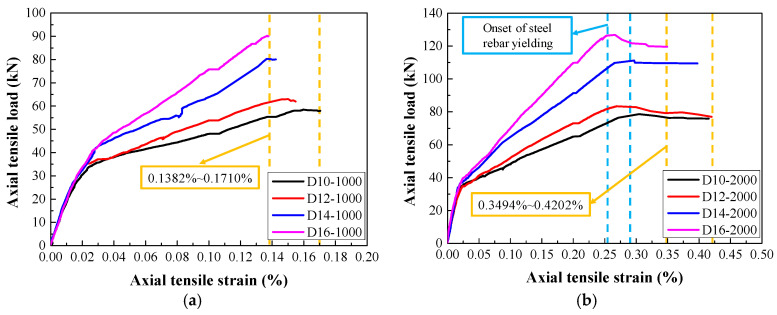

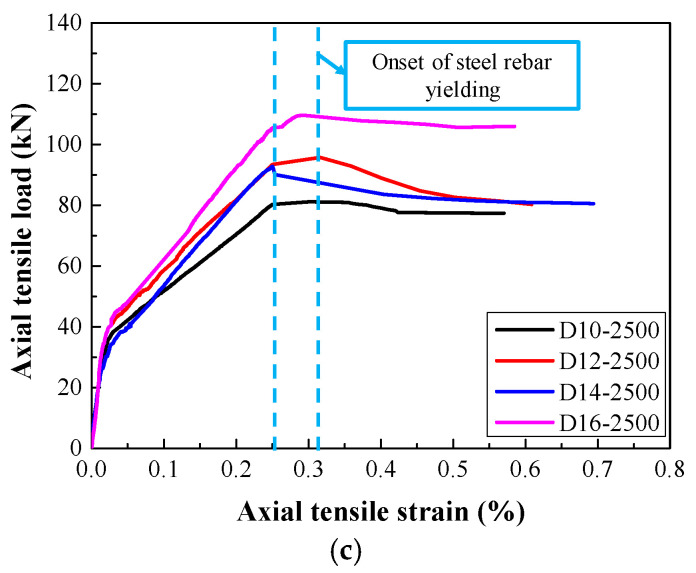
The load–strain envelope curves of steel-reinforced HSHUHPC specimens under cyclic axial tension with different target strains at each cycle: (**a**) 1000 με; (**b**) 2000 με; (**c**) 2500 με.

Figure 10 shows the envelope curves of steel-reinforced HSHUHPC specimens under cyclic tensile loading with different target strain levels in each cycle. As shown in Figure 10a, when the level of the target strain for the tested specimens was 1000 με, the maximum accumulative tensile strain, which floated in the range of 1382–1710 με, did not exceed either the yield strain of the steel rebar or the ultimate tensile strain of HSHUHPC. As shown in Figure 10b, when the level of the target strain for the tested specimens was 2000 με, the maximum accumulative tensile strain floated in the range of 3494–4202 με. By contrast, when the level of the target strain for the tested specimens was 2500 με, it exhibited a slightly larger scatter. Meanwhile, it is observed that the larger the diameter of the embedded steel rebar, the smaller the maximum accumulative tensile strain, which indicated that the larger the diameter of the embedded steel rebar, the greater the contribution to the stiffness of steel-reinforced HSHUHPC specimens in the elastic–plastic stage.

### 3.4. Residual Strain

**Figure 11:**
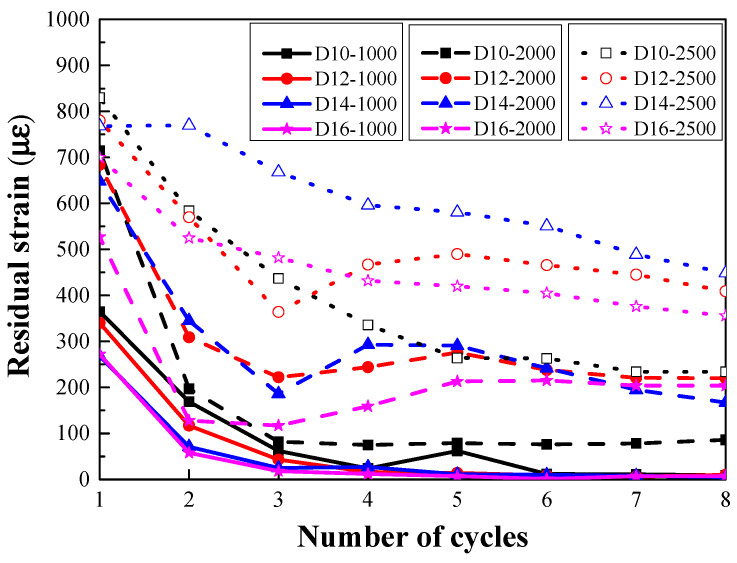
Residual strain at each cycle.

As shown in Figure 11, when the level of the target strain was 1000 με, the residual strain of the steel-reinforced HSHUHPC specimens gradually reduced to the extent of being negligible as the number of cycles increased, because the residual deformation gradually reduced during the loading process under the constant target strain. New damage was generated only when the target strain of reloading exceeded the cumulative strain. On the other hand, the reinforcement was still in the elastic state, and HSHUHPC was in the strain hardening state in the present case; thus the specimen exhibited uniformly distributed micro-cracks, invisible to the eyes.

When the level of the target strain was 2000 με and 2500 με, the residual strain of steel-reinforced HSHUHPC specimens gradually remained stable once the steel rebar yielded as the number of cycles increased. Upon the yielding of the steel rebar, the crack pattern of the steel-reinforced HSHUHPC transformed the uniformly distributed micro-cracks into a localized single macro-crack. Meanwhile, once the crack width progressed beyond a certain threshold, the role of the bridge effect of fibers in the cracks was enhanced and gradually undermined due to the debonding and pull-out of the fibers.

### 3.5. Stiffness Degradation

**Figure 12:**
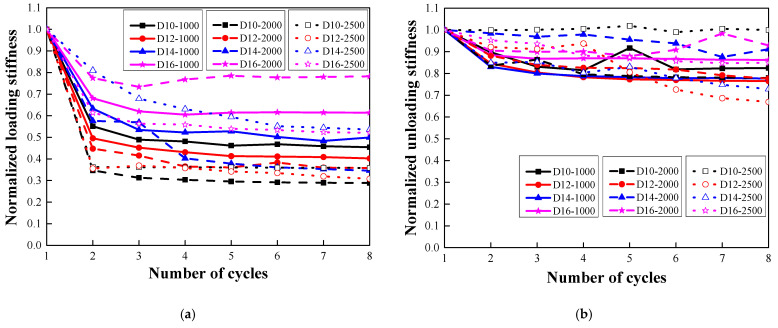
Evolution of tensile stiffness: (**a**) loading stiffness; (**b**) unloading stiffness.

For all the steel-reinforced HSHUHPC specimens, it could be observed that the size of the embedded rebars had a positive effect on the tensile stiffness of the specimens in the elastic stage. Besides, in the case of the elastic–plastic stage, increasing the diameter of the steel rebar upgrades the tensile stiffness effectively. Figure 12 shows the normalized loading stiffness and the normalized unloading stiffness evolution of steel-reinforced HSHUHPC specimens as the number of cycles increases, where the normalized loading stiffness of each cycle refers to the ratio of the loading stiffness at the *i*-th cycle to the initial loading stiffness, and in the same way, the normalized unloading stiffness of each cycle refers to the ratio of the unloading stiffness at the *i*-th cycle to the initial unloading stiffness. These reflect the stiffness degradation degree of the members. Regardless of the level of the target strain, the normalized loading stiffness and unloading stiffness were considerably reduced during the 2nd or 3rd cycle and remained stable with some fluctuations as the number of cycles increased. Compared with Figure 11, the evolution law of the normalized loading stiffness, as the number of cycles increased, was elementarily in accordance with that of the residual strain.

### 3.6. Damage Evolution Process Based on AE Locating

**Figure 13:**
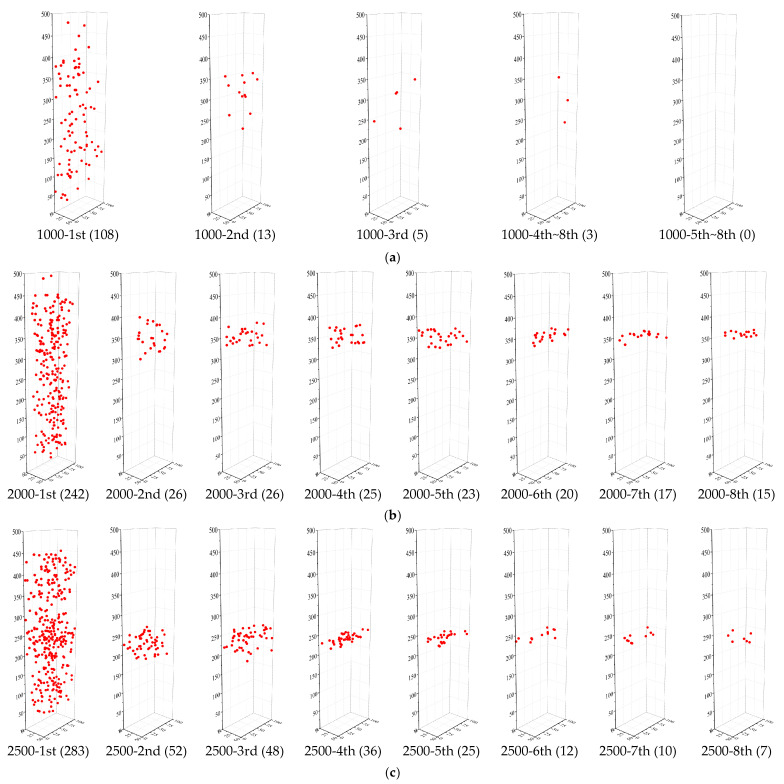
AE source distribution of some typical specimens: (**a**) D12-1000; (**b**) D16-2000; (**c**) D14-2500.

AE analysis technology could effectively monitor and detect the internal damages of UHPC under cyclic tensile loading on the microcosmic level. As mentioned above, the diameter of the embedded steel rebar has little influence on the damage pattern; thus a typical specimen for each level of target strain was discussed herein. Additionally, in order to simplify the analysis, the research took 8 cycles for in-depth analysis due to the fact that the experimental phenomena showed that after the 4th cycle, the loading and unloading curves would tend to be stable for all the specimens. Figure 13a shows the AE source distribution of specimen D12-1000. The values in brackets are the numbers of AE sources generated by each cycle that could be registered by the AE analysis system. In the 1st cycle, the AE sources were randomly distributed throughout the specimen at the target strain value of 1000 με. During the following cycles, fewer new AE sources were detected and widely distributed, which indicated multiple damage behaviors in the steel-reinforced HSHUHPC members, whereas no new AE sources were generated after the 5th cycle subsequently. Figure 13b,c shows the AE source distribution of specimens D16-2000 and D14-2500, respectively. As described above, in the 1st cycle, the AE sources were homogeneously distributed along the height of the specimen, reflecting that multiple micro-cracks (or defects) were distributed all over the specimen, which indicated that steel-reinforced HSHUHPC was under a globally uniform stress state. In the 2nd cycle, both specimens exhibited evenly distributed AE sources in a relatively small region. Then, in the following cycles, AE sources were regularly distributed on the same plane subsequently due to the fact that the embedded steel rebar reached the yield that was almost consistent with the actual location of macro-cracks. This indicates that the AE source locating technology provides strong evidence for the damage distribution of the whole loading process. By contrast, the analysis of AE parameters such as frequency and energy, which are used to characterize the damage mode and damage degree, would not be described in this paper.

## 4. Discussion

### 4.1. Tension-Stiffening Effect

**Figure 14:**
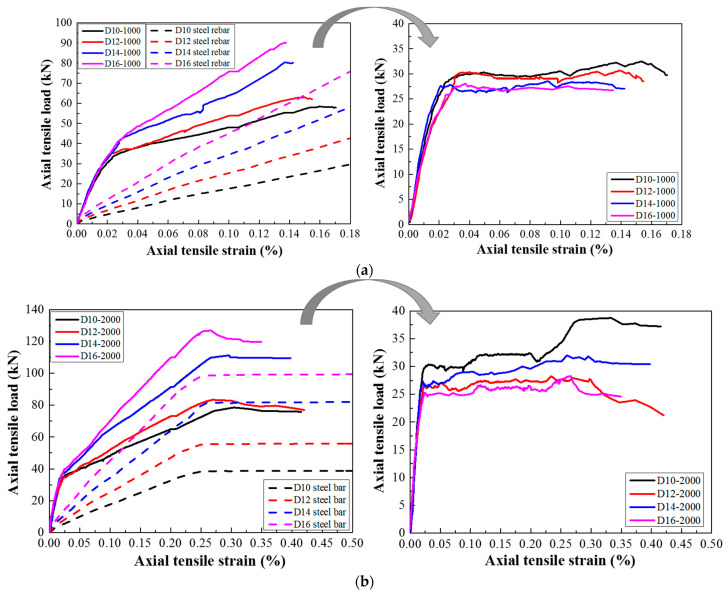
Tension-stiffening responses of the steel-reinforced HSHUHPC specimens under cyclic axial tension with different target strain levels: (**a**) 1000 με; (**b**) 2000 με.

The total tensile bearing capacity of steel-reinforced HSHUHPC consists of steel rebar and HSHUHPC. By getting rid of the pure steel rebar response from the total tensile load–strain envelope curve, the contributions of HSHUHPC in the steel-reinforced HSHUHPC member, usually called the tension-stiffening effect, are obtained as shown in Figure 14. It can be observed in Figure 14 that the tension-stiffening responses for steel-reinforced HSHUHPC members were similar despite variations in the target strain in each cycle. Meanwhile, the reductions in the tensile strength of UHPC, compared with the tested direct tensile strength of HSHUHPC, were most likely due to the localized stress concentration of UHPC around the ribs and the presence of discontinuous fiber distribution in HSHUHPC. Moreover, it was found that a larger embedded steel rebar (namely, a higher steel reinforcing ratio) appeared to reduce the peak tensile strength of the HSHUHPC. By contrast, there are instances where conflicting results on the tension-stiffening response of reinforced UHPC are reported by Hung et al. [11]. This might be because with a larger diameter of the steel rebar, although the contact area between the steel rebar and UHPC increases, the probability of discontinuous distribution of the steel fiber at the interface between the steel rebar and UHPC is greater, and despite the presence of autogenous shrinkage of UHPC, the bond interaction at the interface between the steel rebar and UHPC is weaker.

In addition, it is hypothesized that steel yields when the concrete crushes in traditional reinforced concrete design methods based on the mechanics of slender beams. It is observed experimentally that an early strain hardening effect in the steel occurs due to the high bond strength between HSHUHPC and steel rebar and the tension-stiffening effect. Therefore, traditional methods based on the mechanics of slender beams might typically underestimate the strength of reinforced HSHUHPC components subjected to bending, even when accounting for the tensile capacity of the HSHUHPC material. Further experimental research, including the effect of reinforcement ratio, layout of reinforcement, and evaluation of the flexural capacity of the UHPC slender beam considering the tension-stiffening effect, need to be done in combination with numerical simulation, theoretical calculation, and so on.

### 4.2. Stiffness Degradation Mechanism

**Figure 15:**
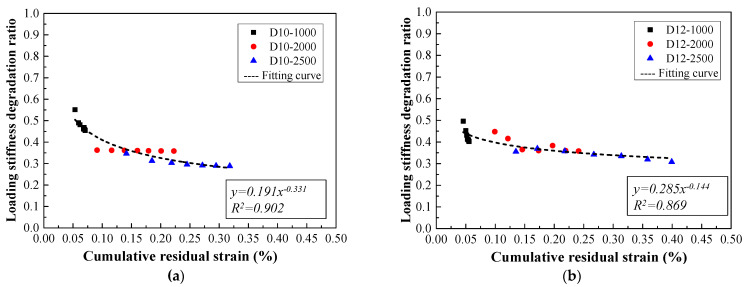

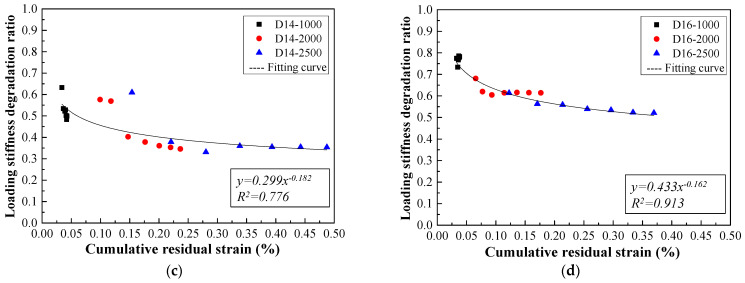
Relationship between the loading stiffness degradation ratio and the cumulative residual strain for different diameters: (**a**) 10 mm; (**b**) 12 mm; (**c**) 14 mm; (**d**) 16 mm.

As mentioned above, it could be supposed that there was hardly a slip generated between HSHUHPC and steel rebar prior to the yielding of the steel rebar. Hence the steel-reinforced HSHUHPC could be regarded as the isotropic material and undergo linear elastic deformation in coordination between steel rebar and HSHUHPC until reached at the ultimate tensile load. According to the research study by Guo Jun-Yuan et al. [22], it is assumed that steel fibers are evenly and continuously distributed in parallel and have the same load direction in HSHUHPC, which is simplified to a chain model. In summary, it is hypothesized that herein both the steel fiber and the steel rebar are arranged evenly in the HSHUHPC members. Therefore, the power function might be used to fit the relationship between the cumulative residual strain and the ratio of loading stiffness degradation based on the conclusions deduced by Guo Jun-Yuan et al. [21]. The relationship between the loading stiffness degradation ratio and the cumulative residual strain at different target strain levels in each cycle is plotted in Figure 15. The relationship model between the loading stiffness degradation ratio and the cumulative residual strain agrees well with the test results with the goodness of fit (R^2^) of 0.902, 0.869, 0.776, and 0.913 for different diameters of the embedded steel rebar. The presence of some large discreteness is due to the great difference in stiffness degradation rate at the elastic stage and the strain-hardening stage, respectively. The relationship model needs to be studied further using more tests.

## 5. Conclusions

Twelve steel-reinforced HSHUHPC specimens under cyclic axial tension, accompanied with AE sources locating technology, were tested in this paper. The effects of the loading pattern, the diameter of the embedded rebar, and the level of target strain in each cycle on the tensile behavior of steel-reinforced HSHUHPC were studied. The following conclusions were drawn:

(1) For a target strain of 1000 με in each cycle, the typical failure of steel-reinforced HSHUHPC exhibited uniformly distributed multiple micro-cracks due to the excellent crack width control ability of HSHUHPC, while for the target strain of 2000 and 2500 με in each cycle, the typical failure pattern exhibited a single, localized macro-crack in conjunction with a sprinkling of narrow and closely spaced micro-cracks due to the inclusion of steel fibers, which intensified the strain concentration in the embedded steel rebar. AE source locating technology provides strong evidence for the damage distribution of the whole loading process.
(2) In terms of load–strain response and envelope curves, the larger the diameter of embedded steel rebar, the smaller the maximum accumulative tensile strain under cyclic tension, which indicated that the larger the diameter of embedded steel rebar, the greater the contribution to the stiffness of the R-HSHUHPC specimens in the elastic–plastic stage.
(3) For the target strain levels of 2000 με and 2500 με in each cycle, once the embedded steel rebar yielded and the crack width progressed beyond a certain threshold, fibers began to pull out, and the ability of the fibers to bridge the cracks began to decrease. Hence the residual strain and stiffness degradation rate in each cycle remained stable.
(4) According to the tension-stiffening response of steel-reinforced HSHUHPC members, the larger the embedded steel rebar, the lower the peak tensile strength of the HSHUHPC after deducting the steel rebar. This is most likely due to the fact that the larger diameter of the steel rebar results in a worse discontinuous distribution of the steel fiber, although the contact area between steel rebar and UHPC increases, and due to the presence of autogenous shrinkage, the bond interaction at the interface between steel rebar and UHPC is weaker.
(5) The relationship model between the loading stiffness degradation ratio and the cumulative residual strain for steel-reinforced HSHUHPC members was proposed. The relationships can be employed to evaluate the stiffness of steel-reinforced HSHUHPC under cyclic tension to some extent.

Further studies are needed to include different fiber volumes, steel rebar diameter-to-cover ratios, and reinforcement ratios to better understand how the mutual contribution of steel fibers and rebars, splitting cracks, and so on influence the damage progression and deformation capacity of the reinforced UHPC. Additional tests are necessary to evaluate the flexural capacity of reinforced UHPC members considering the tension-stiffening effect.

## Figures and Tables

**Table 1:** Specimen details.

Specimen	Diameter of Embedded Rebar (mm)	Loading Pattern	Target Strain (με)
R-HSHUHPC-D10	10	monotonic tension	25,000
R-HSHUHPC-D12	12	monotonic tension	25,000
R-HSHUHPC-D14	14	monotonic tension	25,000
R-HSHUHPC-D16	16	monotonic tension	25,000
D10-1000	10	cyclic tension	1000
D10-2000	10	cyclic tension	2000
D10-2500	10	cyclic tension	2500
D12-1000	12	cyclic tension	1000
D12-2000	12	cyclic tension	2000
D12-2500	12	cyclic tension	2500
D14-1000	14	cyclic tension	1000
D14-2000	14	cyclic tension	2000
D14-2500	14	cyclic tension	2500
D16-1000	16	cyclic tension	1000
D16-2000	16	cyclic tension	2000
D16-2500	16	cyclic tension	2500

**Table 2:** Mix proportions of UHPC material (proportion by weight).

Cement	Silica Fume	Fine Filler	Fine Aggregate	Water	Superplasticizer
1	0.3	0.3	1.34	0.2	0.005

**Table 3:** Properties of steel fibers.

Fiber Type	$f_{y}$ (MPa)	$E_{s}$ (GPa)	*l* (mm)	*d* (μm)	*μ*	Density (kg·m^−3^)
steel fiber	2500	200	16	200	80	7850

Note: $f_{y}\;$= tensile strength; $E_{s}\;$= elastic modulus; *l* = length; *d* = diameter; *μ* = aspect ratio.

## Data Availability

The data presented in this study are available within the article.
